# Space–time clustering of childhood high hyperdiploid B-cell precursor acute lymphoblastic leukemia: a nationwide Swedish study

**DOI:** 10.1007/s10654-025-01323-9

**Published:** 2026-01-12

**Authors:** Gleb Bychkov, Niklas Engsner, Benedicte Bang, Mats Marshall Heyman, Gisela Barbany, Anna Skarin Nordenvall, Giorgio Tettamanti, Claes Strannegård, Ann Nordgren

**Affiliations:** 1 Department of Molecular Medicine and Surgery, Center for Molecular Medicine, Karolinska Institutet, Stockholm, Sweden; 2 Department of Women’s and Children’s Health, Karolinska Institutet, Stockholm, Sweden; 3 Department of Clinical Genetics and Genomics, Karolinska University Hospital, Stockholm, Sweden; 4 Department of Radiology, Karolinska University Hospital, Stockholm, Sweden; 5 Unit of Epidemiology, Institute of Environmental Medicine, Karolinska University Hospital, Stockholm, Sweden; 6 Department of Applied Information Technology, University of Gothenburg, Gothenburg, Sweden; 7 Institute of Biomedicine, Department of Laboratory Medicine, University of Gothenburg, Gothenburg, Sweden; 8 Department of Clinical Genetics and Genomics, Sahlgrenska University Hospital, Region Västra Götaland, Gothenburg,, Sweden

**Keywords:** Lymphoblastic, leukemia, acute, Child, Space–time clustering analysis, High hyperdiploid, ETV6:RUNX1

## Abstract

Acute lymphoblastic leukemia (ALL) is the most common childhood malignancy. While space–time clustering of ALL cases has been suggested, only one prior study has examined clustering by genetic subtype. We investigated space–time clustering of childhood ALL in Sweden, both overall and by genetic subtype. The cohort included 1,629 children age 0–18 years diagnosed with ALL between 1992 and 2017, comprising 1,446 B-cell precursor ALL (BCP-ALL) and 183 T-cell ALL (T-ALL) cases. Two BCP-ALL subgroups were analyzed: high hyperdiploidy (HeH, *n* = 466) and *ETV6::RUNX1* (*n* = 225). The Unbiased Knox Test and Unbiased Combined Knox Test were used to assess space–time clustering at the municipality level, accounting for multiple testing and population shifts. The Density-Based Spatial Clustering of Applications with Noise (DBSCAN) algorithm was applied to identify significant clusters. Logistic regression was used to evaluate demographic differences between clusters, including age, sex, and birth order. Significant space–time clustering was observed in the HeH subgroup for both place and date of birth (*p* = 0.005) and place and date of diagnosis (*p* = 0.011), at space–time thresholds of 40 km/18 months and 30 km/24 months, respectively. No clustering was detected in the overall BCP-ALL group, T-ALL group, or the *ETV6::RUNX1* subgroup. Space–time clustering at birth and diagnosis was observed in the HeH subgroup, suggesting potential etiologic heterogeneity in BCP-ALL. These findings support further investigation of environmental and infectious exposures across immunophenotypes and genetic subtypes in larger cohorts.

**Supplementary Information:**

The online version contains supplementary material available at 10.1007/s10654-025-01323-9.

## Introduction

Acute lymphoblastic leukemia (ALL) is the most common childhood malignancy, accounting for approximately one-third of all pediatric malignancies worldwide [1]. B-cell precursor acute lymphoblastic leukemia (BCP-ALL) is the predominant subtype, representing approximately 85% of cases [2]. Over the last decades, survival for children with BCP-ALL has improved markedly, with overall survival now exceeding 90% in high-income countries, largely due to advances in therapy and supportive care. Nevertheless, a substantial proportion of patients experience severe long-term treatment-related side effects, and disparities in survival persist based on age at diagnosis, ethnicity, socioeconomic status, genetic predisposition, immunophenotype, and genetic subtype of leukemia [3–5].

To date, 24 genetically distinct subtypes of BCP-ALL have been characterized [4, 6]. Among these, 11 genetic subtypes were recently included in the inaugural 2022 WHO Classification of Pediatric Tumors [2]. Identifying genetic subtypes in childhood ALL is essential, as some convey prognostic significance, while others present opportunities for targeted therapy.

The two most common subtypes in children are high hyperdiploid (HeH) ALL, characterized by the presence of extra chromosomes (typically 51–67), and *ETV6::RUNX1-*positive ALL, resulting from the t(12;21)(p13;q22) translocation. Collectively, HeH and *ETV6::RUNX1* account for approximately 50% of childhood BCP-ALL cases, and both are generally considered favorable-prognostic subtypes [7]. In childhood BCP-ALL, the initial genetic event - such as *ETV6::RUNX1* or HeH - often arises in utero and can be detected as a preleukemic clone in neonatal blood spots. Population-based studies suggest that approximately 1–5% of healthy newborns harbor the *ETV6::RUNX1* fusion. However, in most cases, these preleukemic clones are effectively controlled or eliminated by the immune system, and the vast majority of carriers never progress to overt leukemia [8, 9].

The etiology of BCP-ALL remains largely unknown, but genetic predisposition, ionizing radiation, environmental toxins, and aberrant training of the child’s immune system are considered contributing factors contributing to leukemogenesis [10–15]. Epidemiological studies that specifically address distinct risk-factor profiles for immunophenotypically and cytogenomically defined leukemia subtypes remain limited [16]. Several studies have reported associations between environmental exposures and certain ALL subtypes, including links between household paint exposure in *KMT2A*-rearrangements and *ETV6::RUNX1* fusion, tea in *KMT2A*-rearrangements and exposure to sunlight in HeH, and pesticides in children with HeH and *ETV6::RUNX1* fusion [16, 17]. The role of common pathogens in the etiology of ALL has been given great interest in both epidemiological and molecular studies. In 1988, two complementary models linked childhood leukemia to aberrant immune responses to common infections. Kinlen’s population-mixing hypothesis proposed that when previously isolated communities are exposed to new infectious agents, a small subset of immunologically naïve children may develop leukemia, as a rare outcome [18]. Greaves’ two-hit model proposes that BCP-ALL originates from prenatal preleukemic clones (first hit), with delayed exposure to common childhood infections in early childhood, triggering dysregulated immune responses and a second genetic hit that drives progression to overt disease [19]. In 1992, Alexander suggested that some children develop a persistent infection after exposure in utero or around the time of birth, which could increase their risk of developing leukemia, especially at an older age of onset. The author suggested that it is not only the infection itself but also the “window” in time when it occurred is central to its ability to promote progression to different subtypes of BCP-ALL [20]. An important feature of childhood ALL is the incidence peak at 2–6 years of age, which coincides with a critical stage of immune maturation and increased exposure to common infections through social contacts. Prevailing “delayed infection” hypothesis proposes that insufficient immune priming early in life, followed by dysregulated responses to later infections, may provide the secondary “hits” that drive progression of preleukemic clones to overt ALL [19–22].

The detection of space–time clustering among BCP-ALL cases in relation to genetic subtype provides valuable insights into the etiology of leukemia. Several studies have reported space–time clustering in ALL [23–28], however only one previous study has incorporated genetic subtypes into the space–time clustering analysis [29]. In the present study, we investigate space–time clustering in a large cohort of childhood ALL, both by immunophenotype and by genetic subtype.

## Materials and methods

**Fig. 1:**
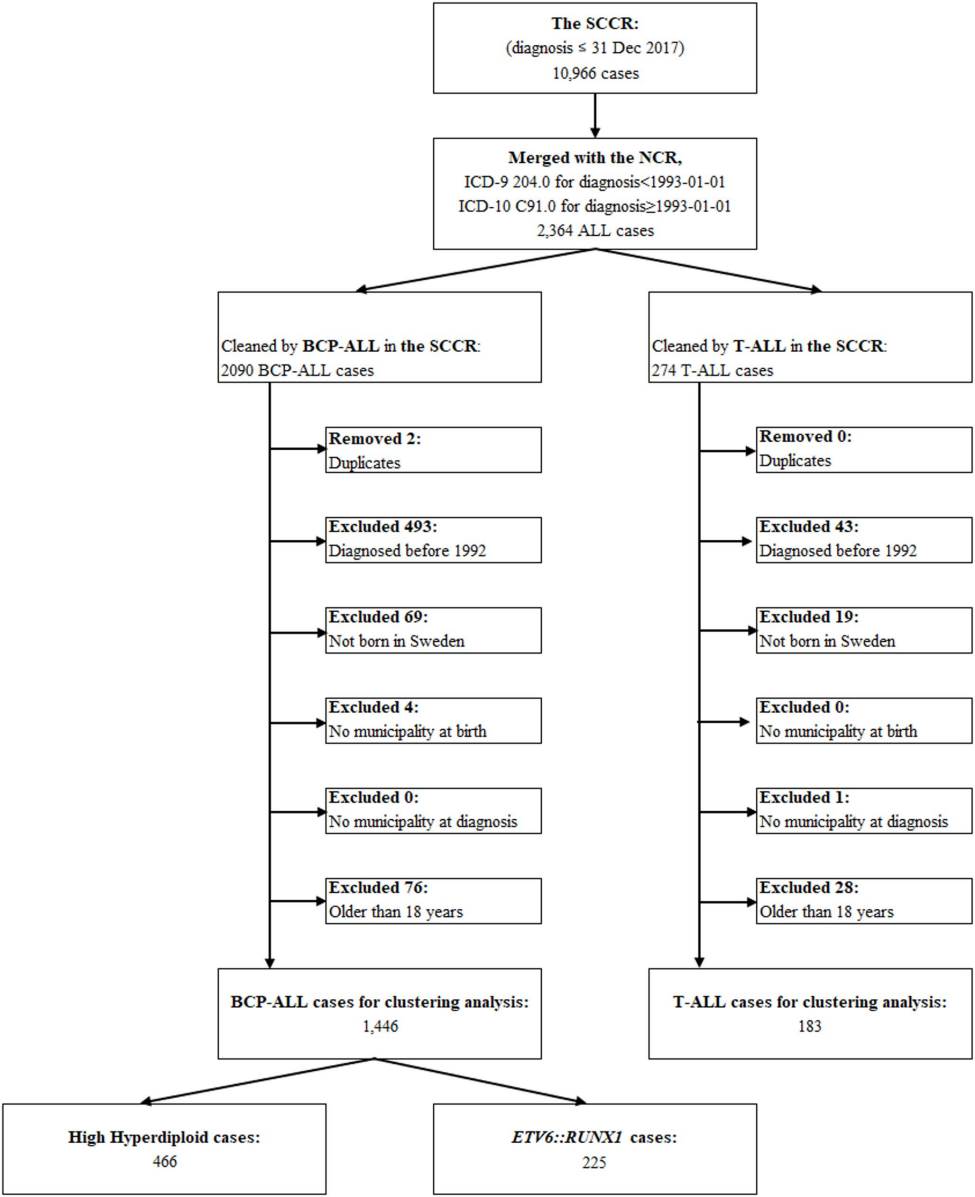
Case selection flow for BCP-ALL, T-ALL, and genetic subtypes (HeH, *ETV6::RUNX1*)

We conducted a nationwide, register-based study in Sweden using data from the Swedish Childhood Cancer Registry (SCCR) [30], the National Cancer Register (NCR) [31], and the Total Population Register (TPR) [32]. Linkage between these national registers was enabled by the personal identity number (PIN), which is assigned to all Swedish residents [33]. The SCCR is a National Quality Registry that has collected data on pediatric tumors and hematological malignancies since the early 1970 s, with consistent reporting from the 1980s. The most common BCP-ALL subtypes, HeH and *ETV6::RUNX1*, have been recorded since 1992 and 2000, respectively, following the introduction of reliable genetic diagnostic methods. The NCR is a nationwide database that has systematically collected and maintained detailed information on cancer diagnoses in Sweden since 1958, including patient demographics (sex and age at diagnosis) and tumor characteristics (topography and morphology codes) [31]. Cancer diagnoses in the NCR were classified using ICD-7 codes from 1958, with transitions to ICD-9 in 1987, ICD-O/2 in 1993 and ICD-O/3 in 2005. The TPR, managed by Statistics Sweden, maintains demographic records for the Swedish population. For this study, TPR data were used to determine the municipality of residence at birth and at the time of ALL diagnosis, as exact addresses were unavailable due to ethical constraints. The pseudonymized data included dates of birth, genetic subtype, and residential municipality at birth and diagnosis (recorded at month–year resolution). The cohort was ascertained through linkage of SCCR and NCR registries, with duplicate entries systematically identified and reconciled. A flow diagram of inclusions and exclusions is shown in Fig. 1. The ICD-7 code 204.0 for ALL recorded in the NCR was used to identify cases. BCP-ALL and T-ALL immunophenotypes, along with BCP-ALL cytogenetic subgroups, were verified against SCCR data, in which these classifications are routinely recorded. Only individuals born and diagnosed in Sweden were included.

### Register data and variables

From the NCR, ALL cases aged 0–18 years were identified using ICD-9 code 204.0 for diagnoses before 1 January 1993 and ICD-10 code C91.0 for diagnoses from 1 January 1993 onward, with a unified ALL definition applied for harmonization. From the SCCR, we obtained data of diagnosis, immunophenotype (BCP-ALL, T-ALL), and genetic subtypes; the HeH subtype has been recorded since 1992 and the *ETV6::RUNX1* subtype since 2000. From the TPR, we obtained sex, date and place of birth, municipality of residence at birth and diagnosis, information about biological parents and siblings (to determine birth order), and municipality-by-month resident population and live-birth counts (used to adjust for population shifts). Genetic-subtype analyses focused on HeH and *ETV6::RUNX1*. Cases with other or unknown genetic subtypes were retained in the overall ALL cohort but excluded from subtype-specific analyses.

### Residential location and geocoding

Locations were represented at the municipality level across Sweden’s 290 municipalities. Each municipality was assigned the latitude and longitude of its municipal seat in WGS 84 [34]. Pairwise distances between municipal seats were computed using the haversine formula and reported in kilometers [35]. Birth-based analyses used municipality of residence at birth; diagnosis-based analyses used municipality of residence at diagnosis.

### Statistical analyses

We assessed space–time interaction using the unbiased Knox framework [36, 37]. The Unbiased Knox Test (K-Test) adjusts for population shifts (uneven growth, migration) via population simulations, yielding p-values for any pre-specified space–time threshold pair. Critical space–time thresholds were pre-specified as a 9 × 6 grid: temporal thresholds of 1, 2, 3, 6, 9, 12, 18, 24, and 30 months and spatial thresholds of 1, 10, 20, 30, 40, and 50 km, selected a priori to align with our month-level date resolution and plausible epidemiologic scales. These windows cover short intervals of infectious spread (~ 1–3 months), seasonality (~ 6–12 months), longer infection-incidence cycles (~ 18–30 months), and distances between neighboring municipalities, consistent with municipality-level geocoding in Sweden. Address-level geocodes and exact dates would enable finer thresholds, very short windows, and models that account for population heterogeneity and mobility.

To identify global clustering without inflating type I error across this grid, we used the Unbiased Combined Knox Test (CK-Test), applying the Kulldorff–Hjalmars modification of Baker’s procedure to control for multiplicity [37, 38]. The CK-Test p-value summarizes evidence over all thresholds. In parallel, the K-Test provides p-values for each threshold pair; we selected the most indicative pair (smallest K-Test p within the grid) and used it to parameterize Density-Based Spatial Clustering of Applications with Noise (DBSCAN) [39] to identify clustered and non-clustered cases.

We applied DBSCAN with a joint space–time neighborhood radius $\:\epsilon\:=\left({\epsilon\:}_{space},\:{\epsilon\:}_{time}\right),\:$where $\:{\epsilon\:}_{space}$ was set to the spatial threshold and $\:{\epsilon\:}_{time}$ to the corresponding temporal threshold from the most indicative K-Test pair. We set minPts = 2, which stipulates that a set of cases must contain at least two points to count as a cluster. Two cases were treated as neighbors if their geodesic distance $\:\le\:{\epsilon\:}_{space}$ and their absolute time difference $\:\le\:{\epsilon\:}_{time}$. DBSCAN then formed clusters as sets of density-connected cases; points not density-reachable from any core case were labeled non-clustered.

We modelled DBSCAN cluster membership as the outcome in a logistic regression. Predictors were sex, age at diagnosis, and birth order. The reference categories were female sex, age 1–4 years, and firstborn. All estimates were reported with 95% CIs; tests were two-sided (α = 0.05).

Further details on the statistical and machine-learning methods are provided in the supplementary material. All analyses were implemented in Python 3.9.

## Results

### Study population

A total of 1,629 children and adolescents diagnosed with ALL before the age of 18, between 1992 and 2017, were included in the study. This cohort comprised 1,446 cases of B-cell precursor ALL and 183 cases of T-cell ALL. Among the BCP-ALL cases, 466 were classified as having the HeH genetic subtype, while 225 were of the *ETV6::RUNX1* subtype (Table 1). A male predominance was seen across groups, most marked in T-ALL (77.6% male) compared with BCP-ALL overall (54.1%), HeH (52.4%), and *ETV6::RUNX1* (59.6%). Age at diagnosis differed by lineage/subtype: the age group 1–4 years comprised the majority of BCP-ALL (53.2%), HeH (65.2%), and *ETV6::RUNX1* (61.8%), whereas T-ALL cases were older (36.1% aged 5–9 and 38.8% aged 10–17), reflected in median ages of 4.3 years (BCP-ALL), 3.9 years (HeH), 4.0 years (*ETV6::RUNX1*), and 8.4 years (T-ALL). By year of diagnosis, BCP-ALL cases were evenly distributed across 1992–1999, 2000–2009, and 2010–2017. HeH cases occurred throughout the study period. *ETV6::RUNX1* cases appeared only from 2000 onward, reflecting the introduction of routine diagnostic testing for this fusion in 2000.

**Table 1:** Baseline demographic and clinical characteristics of the cohort by ALL lineage and BCP-ALL genetic subtype

	BCP-ALL	HeH	*ETV6::RUNX1*	T-ALL
N	1,446	466	225	183
Sex				
Male	782 (54.1)	244 (52.4)	134 (59.6)	142 (77.6)
Female	664 (45.9)	222 (47.6)	91 (40.4)	41 (22.4)
Age at diagnosis				
<1	49 (3.4)	3 (0.6)	2 (0.9)	1 (0.5)
1–4	769 (53.2)	304 (65.2)	139 (61.8)	45 (24.6)
5–9	361 (25.0)	108 (23.3)	73 (32.4)	66 (36.1)
10–17	267 (18.4)	51 (10.9)	11 (4.9)	71 (38.8)
Mean age (SD)	5.9 (4.3)	5.1 (3.6)	4.8 (2.6)	8.7 (4.6)
Median age	4.3	3.9	4	8.4
Year at diagnosis				
1992–1999	464 (32.1)	141 (30.3)	0 (0.0)	58 (31.7)
2000–2009	519 (35.9)	161 (34.5)	105 (46.7)	67 (36.6)
2010–2017	463 (32.0)	164 (35.2)	120 (53.3)	58 (31.7)
Year at birth				
1975–1984	70 (4.8)	16 (3.4)	0 (0.0)	19 (10.4)
1985–1994	464 (32.1)	120 (25.8)	10 (4.4)	76 (41.5)
1995–2004	475 (32.8)	171 (36.7)	91 (40.4)	50 (27.3)
2005–2017	437 (30.3)	159 (34.1)	124 (55.2)	38 (20.8)

BCP-ALL is the total cohort that includes all BCP-ALL cases. Two BCP-ALL subtypes: HeH and *ETV6::RUNX* are reported in the separate columns. Percentage of cohort by column in parentheses, %

### Space–time clustering

Evidence of clustering was observed for the HeH subtype (birth: *p* = 0.005; diagnosis: *p* = 0.011), with p-values adjusted for population shifts and multiplicity across the prespecified threshold grid. No significant clustering was observed for BCP-ALL overall, T-ALL, or the *ETV6::RUNX1* fusion subtype at either birth (place and date) or diagnosis (place and date) (Table 2).

**Table 2:** CK-Test results for space–time clustering by cohort

Cohort	*N*	CK-Test *p* (birth)	CK-Test *p* (diagnosis)
BCP-ALL	1,446	0.871	0.559
HeH	466	0.005	0.011
*ETV6::RUNX1*	225	0.373	0.461
T-ALL	183	0.957	0.25

The p-values were obtained from 999 population simulations under the unbiased Knox null (upper-tail, rank-based). CK-Test p-values for birth-based (place & date of birth) and diagnosis-based (place & date of diagnosis) analyses

The most indicative pairs were 40 km/18 months at birth (*p* = 0.001) and 30 km/24 months at diagnosis (*p* = 0.001) (Table 3). These were the threshold pairs with the largest normalized excess of close pairs, and they yielded the smallest K-Test p-values. The CK-Test summarized the maximum normalized excess across the grid of critical thresholds and produced a single global p-value. Accordingly, the most indicative K-Test threshold was consistent with the CK-Test result. All other threshold-specific p-values were reported for completeness and were not used for inference because they were not adjusted for multiplicity.

**Table 3:** K-Test results for space–time clustering in the heh cohort

	Place/Date of Birth									
	Temporal Threshold (months)									
		1	2	3	6	9	12	18	24	30
Spatial Thresholds (km)	1	0.452 (0.13)	0.521 (−0.09)	0.383 (0.29)	0.44 (0.18)	0.478 (0.11)	0.396 (0.26)	0.168 (1.06)	0.145 (1.2)	0.384 (0.37)
	10	0.291 (0.58)	0.253 (0.65)	0.275 (0.61)	0.225 (0.8)	0.277 (0.66)	0.175 (1.06)	0.052 (2.01)	0.05 (2.04)	0.155 (1.18)
	20	0.436 (0.18)	0.668 (−0.43)	0.319 (0.42)	0.226 (0.73)	0.135 (1.22)	0.124 (1.28)	0.019 (2.63)	0.023 (2.45)	0.06 (1.85)
	30	0.184 (0.96)	0.528 (−0.1)	0.275 (0.55)	0.058 (1.69)	0.014 (2.43)	0.005 (3.09)	0.001 (4.08)	0.001 (3.75)	0.001 (3.44)
	40	0.258 (0.71)	0.521 (−0.06)	0.256 (0.64)	0.052 (1.78)	0.013 (2.51)	0.003 (3.46)	0.001 (4.44)	0.001 (4.12)	0.001 (3.83)
	50	0.192 (0.94)	0.5 (0.01)	0.29 (0.54)	0.134 (1.28)	0.034 (2.05)	0.004 (3.22)	0.002 (3.8)	0.001 (3.69)	0.002 (3.59)

	Place/Date of Birth									
	Temporal Threshold (months)									
		1	2	3	6	9	12	18	24	30
Spatial Thresholds (km)	1	0.227 (0.77)	0.388 (0.3)	0.612 (−0.23)	0.464 (0.19)	0.368 (0.49)	0.06 (1.9)	0.147 (1.26)	0.212 (0.94)	0.141 (1.28)
	10	0.297 (0.59)	0.213 (0.91)	0.445 (0.22)	0.273 (0.76)	0.146 (1.28)	0.048 (2.22)	0.072 (1.88)	0.087 (1.76)	0.111 (1.62)
	20	0.403 (0.2)	0.124 (1.26)	0.471 (0.12)	0.585 (−0.13)	0.414 (0.34)	0.131 (1.29)	0.128 (1.41)	0.074 (1.9)	0.09 (1.81)
	30	0.228 (0.74)	0.062 (1.62)	0.425 (0.27)	0.062 (1.66)	0.023 (2.3)	0.003 (3.72)	0.001 (3.9)	0.001 (4.15)	0.003 (4.03)
	40	0.29 (0.58)	0.143 (1.15)	0.668 (−0.35)	0.037 (2.0)	0.012 (2.65)	0.004 (3.55)	0.005 (3.91)	0.003 (4.03)	0.007 (3.99)
	50	0.429 (0.16)	0.168 (1.03)	0.657 (−0.35)	0.023 (2.13)	0.014 (2.61)	0.005 (3.51)	0.006 (3.68)	0.003 (3.97)	0.004 (4.07)

Threshold specific K-Test p-values with the normalized excess of close pairs in parentheses

### Localized clusters (DBSCAN)

Using the most indicative threshold pairs from the K-Test (HeH: birth 40 km/18 months; diagnosis 30 km/24 months), we applied DBSCAN to classify cases as clustered (core or border) or non-clustered (noise).

For the diagnosis-based analysis of the HeH cohort, DBSCAN identified 48 clusters using a space–time threshold of 40 km and 18 months, grouping 308 cases and identifying 158 cases as outliers (non-clustered cases). The five largest clusters contained 107, 33, 21, 12, and 11 cases (≈ 60% of clustered cases). Among the remaining 43 clusters, sizes ranged from 2 to 8 cases.

In the HeH cohort, at the place and date of diagnosis, DBSCAN identified 47 clusters using temporal and spatial thresholds of 30 km and 24 months. The five largest clusters contained 106, 46, 22, 16, and 11 cases (≈ 64% of clustered cases). Among the remaining 42 clusters, sizes ranged from 2 to 5 cases.

**Fig. 2:**
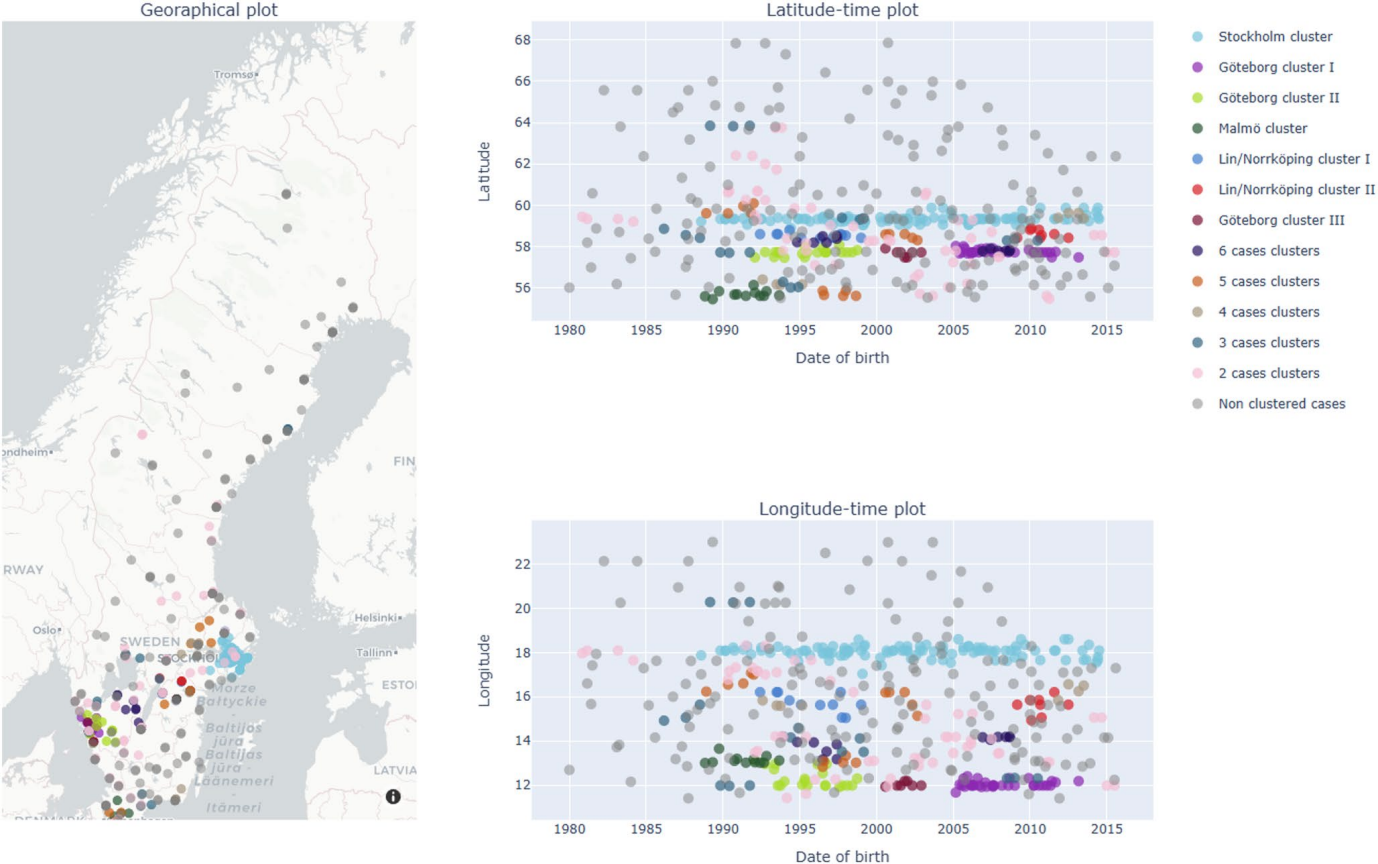
DBSCAN results for HeH at birth (ε_space = 40 km; ε_time = 18 months)

**Fig. 3:**
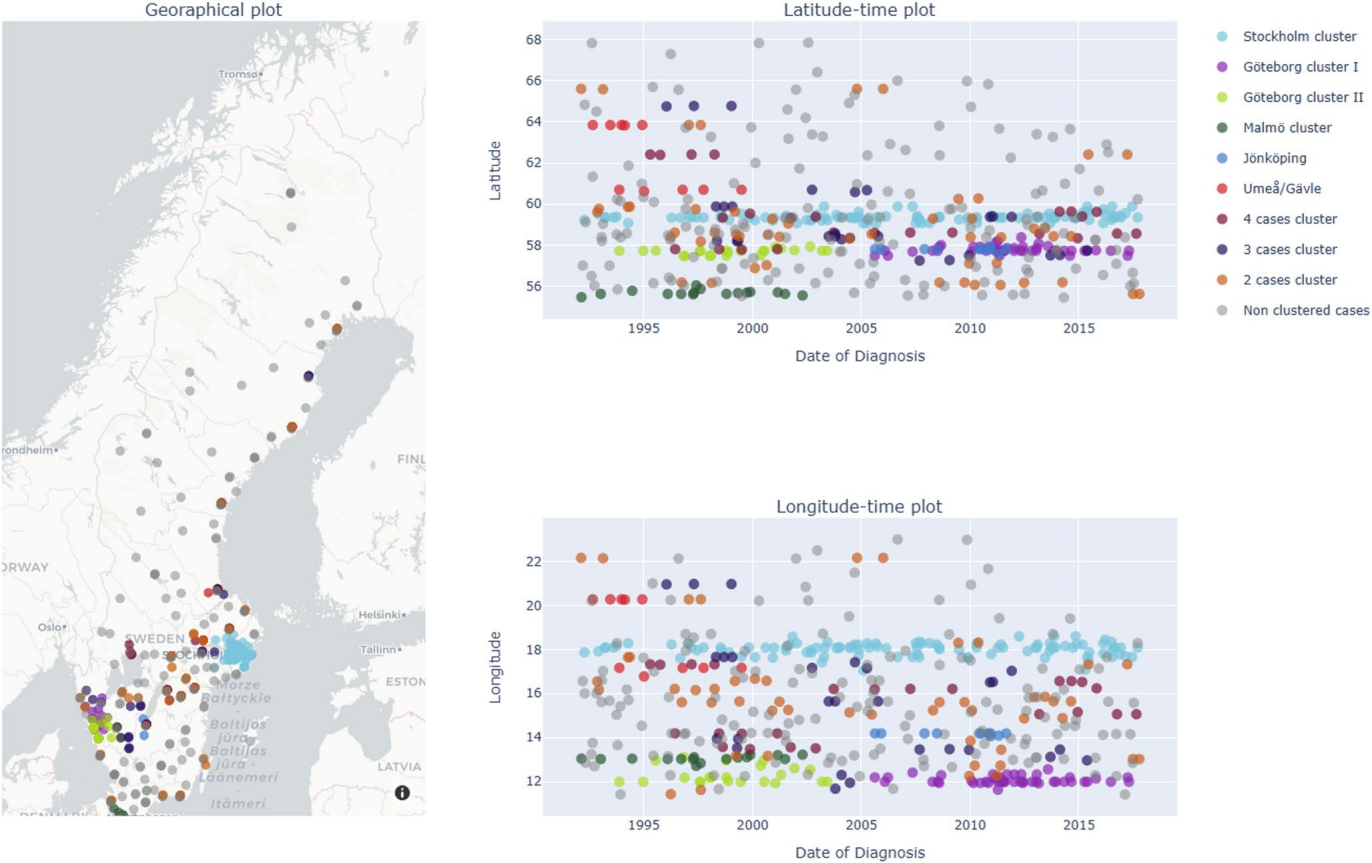
DBSCAN results for HeH at diagnosis (ε_space = 30 km; ε_time = 24 months)

The space–time clustering of HeH cases at birth and at diagnosis is shown in Figs. 2 and 3, respectively.

### Factors associated with clustering

In the birth-based analysis (ε_space = 40 km; ε_time = 18 months), males had lower odds of being in a cluster than females (OR = 0.63; 95% CI 0.43–0.94; *p* = 0.02). Neither age at diagnosis nor birth order was associated with cluster membership (all *p* > 0.05). In the diagnosis-based analysis (ε_space = 30 km; ε_time = 24 months), none of the variables (sex, age, birth order) were associated with clustering (Table 4).

**Table 4:** Logistic regression results for DBSCAN-defined cluster membership in HeH at birth and at diagnosis

Place/Date of birth					
	Clustered	Non clustered	OR	CI	*P*-value
Cases	308	158			
Characteristics					
Sex (Male)	150/308 (48.7)	94/158 (59.5)	0.63	[0.43, 0.93]	0.02
Age					
< 1	2/308 (0.6)	1/158 (0.6)	0.76	[0.07, 8.77]	0.83
1–4	209/308 (67.9)	95/158 (60.1)			
5–9	69/308 (22.4)	39/158 (24.7)	0.75	[0.47, 1.22]	0.25
10–17	28/308 (9.1)	23/158 (14.6)	0.6	[0.34, 1.07]	0.08
Birth Order					
1	235/308 (76.3)	117/158 (74.1)			
2	19/308 (6.2)	12/158 (7.6)	0.75	[0.35, 1.62]	0.47
3	35/308 (11.4)	16/158 (10.1)	1.07	[0.57, 2.04]	0.82
> 3	19/308 (6.2)	13/158 (8.2)	0.7	[0.33, 1.48]	0.35

Place/Date of diagnosis					
	Clustered	Non clustered	OR	CI	*P*-value
Cases	312	154			
Characteristics					
Sex (Male)	155/312 (49.7)	89/154 (57.8)	0.7	[0.47, 1.04]	0.08
Age					
< 1	2/312 (0.6)	1/154 (0.6)	1	[0.09, 11.93]	0.99
1–4	206/312 (66.0)	98/154 (63.6)			
5–9	71/312 (22.8)	37/154 (24.0)	1	[0.52, 1.35]	0.46
10–17	33/312 (10.6)	18/154 (11.8)	1.0	[0.55, 1.82]	0.99
Birth Order					
1	236/312 (75.6)	116/154 (75.3)			
2	20/312 (6.4)	11/154 (7.1)	0.88	[0.41, 1.91]	0.75
3	40/312 (12.8)	11/154 (7.1)	1.82	[0.9, 3.69]	0.1
> 3	16/312 (5.1)	16/154 (10.5)	0.48	[0.23, 1.0]	0.051

## Discussion

We performed space–time clustering analysis in a Swedish cohort of 1,629 ALL cases and identified space–time clustering in the HeH subgroup, both at birth and at diagnosis (place and date), within the most indicative space–time window of 40 km/18 months, 30 km/24 months, respectively. To the best of our knowledge, this is the first study to assess spatiotemporal clustering of a genetic subtype of BCP-ALL at the national level.

BCP-ALL is a genetically diverse disease, and different environmental and genetic factors are likely to contribute to the development of its subtypes. However, most previous studies on space–time clustering have focused on large, mixed groups of BCP-ALL cases, which may have masked the detection of clustering specific to genetic subtypes. Space–time clustering studies are observational and can suggest potential etiologic factors, such as common exposures or conditions, that can be further explored.

Two previous studies by Gustafsson and Carstensen investigated possible space–time clustering of childhood ALL in Sweden. The first cohort consisted of 609 children diagnosed with ALL between 1973 and 1989 [40]. In the extended study, 1,024 ALL cases (0–14 years) diagnosed between 1973 and 1996 were included [41]. Knox’s method for the detection of space–time clustering was used in both studies, and a significant excess of case pairs close in time and space at birth was found, while no statistically significant clustering around the time of diagnosis was detected. Compared to these previous studies on space–time clustering of childhood leukemia in Sweden, we have been able to incorporate immunophenotyping, extend the study period, include genetic subtypes of BCP-ALL, and take migration into account in our analysis. Some of these advances were enabled by improvements in the completeness and quality of Swedish registries in recent decades.

Several studies have applied the Knox test to evaluate space–time clustering of childhood leukemia [20, 40–46], but these analyses did not stratify by immunophenotype or genetic subtype. Since BCP-ALL and T-ALL differ in age profiles and may have partly distinct etiologies, pooling across them can dilute or obscure disease-specific characteristics. In contrast, McNally et al. performed a study based on immunophenotypes and reported significant clustering with respect to time and place of birth for BCP-ALL within the age group 18–54 months, whereas aggregate analyses did not show the same pattern [47]. The first study to include genetic subtypes in the space–time clustering analysis of ALL was performed by Kreis et al. [29], who reported a statistically significant prevalence of the *ETV6::RUNX1* genetic subtype in clustered cases compared with non-clustered cases in Switzerland. The authors utilized the K-Test to identify clusters and logistic regression to estimate the significant prevalence of the *ETV6::RUNX1* genetic subtype. We did not observe space–time clustering of the *ETV6::RUNX1* subtype in our study; instead, we found clustering of the HeH subtype. Several factors could account for this difference, including population characteristics, sample size, study period, climate, and patterns of infectious exposure. One of the advantages of the Kreis et al. study is access to precise residential geolocation data for all cases included in the study, whereas our analyses were restricted to the municipality level. However, Kreis et al. did not perform the K-Test or CK-Test for a specific subtype; instead, they assessed the prevalence of the *ETV6::RUNX1* subtype in the significant clusters of BCP-ALL. One advantage of our study is the larger cohort size (466 vs. 109 HeH cases and 225 vs. 93 *ETV6::RUNX1* cases), which enabled direct application of the K-Test and CK-Test in the genetic subtypes.

The HeH subtype comprises 25–30% of all BCP-ALLs and is characterized by non-random gains of chromosomes, predominantly X, 4, 6, 10, 14, 17, 18, and 21. The first preleukemic event resulting in HeH often occurs before birth, although overt leukemia does not manifest until several years later [48–50]. For a long time, it has been largely unknown how aneuploidy arises, but recently, Woodward et al. [51] conducted single-cell whole-genome sequencing (scWGS) and demonstrated stable chromosomal content of HeH leukemic cells, suggesting that HeH likely arises from an initial tripolar mitosis in a diploid cell early in leukemogenesis. Tripolar mitosis occurs in cancer cells and may be induced by multiple factors, such as viral infections [52–54], mitotic spindle toxins [55, 56], and irradiation [57].

Our finding of space–time clustering of HeH cases at birth does not allow definitive conclusions about underlying causes, but it does suggest that a distinct mechanism operating during fetal development may generate the pre-leukemic HeH clone. In embryonic cells, the cellular stress capable of triggering tripolar mitoses can arise from both intrinsic factors, such as replication or metabolic stress, and extrinsic influences that disrupt normal cell-cycle regulation. Potential contributors include environmental exposures and infectious agents, some of which may exhibit seasonal or other periodic patterns, providing a biologically plausible explanation for the observed clustering. The clusters in our study were concentrated in large cities, which may reflect the higher proportion of immigrants residing in urban areas of Sweden and aligns with the Kinlen hypothesis that increased population mixing can promote localized childhood leukemia risk through exposure to novel infections [18].

To date, no molecular evidence of a disease-initiating infectious agent, such as the HPV for cervical carcinoma [58] and the EBV for Burkitt’s Lymphoma [59], has been identified for ALL. To clarify the etiology of HeH BCP-ALL and evaluate potential in utero viral contributions, maternal and cord-blood sera could be analyzed using high-throughput sequencing and serologic assays. However, direct fetal infection is not necessarily required since maternal immune or inflammatory responses also may release mediators that cross the placenta and disrupt fetal hematopoiesis. Additional insight could be obtained by comparing the presence, genomic distribution, and integration patterns of viral sequences in germline DNA and in leukemic blasts from affected patients. A “hit-and-run” mechanism, in which transient maternal viral exposure triggers pathogenic changes without persistent infection, also remains plausible.

To our knowledge, this is the first study to identify significant clustering of HeH at the time of diagnosis. However, caution is warranted in interpreting this finding. Analyses based on birth and diagnosis yielded comparable CK-Test p-values, similar indicative thresholds, and similar proportions of clustered versus non-clustered cases. Given the young median age at diagnosis (~ 4 years) and low residential mobility (only 13% of HeH cases changed municipality between birth and diagnosis), the space–time distribution of HeH cases appeared largely unchanged from birth to diagnosis. Furthermore, our month–year dating and municipality-level geocoding preserve alignment between birth and diagnosis locations, even for moves within a municipality, which may bias towards similar clustering signals at both time points. Consequently, the clustering observed at diagnosis is likely confounded by the clustering observed at birth; we therefore interpret the finding of clustering at diagnosis as supportive but not independent of birth-time clustering.

We observed a strong correspondence between HeH clustering at birth and at diagnosis, which limits the interpretability of other cross-combinations (date of birth × place of diagnosis; date of diagnosis × place of birth). Although such analyses have been reported previously [46, 60], we therefore focused on the two primary formulations: time/place of birth and time/place of diagnosis.

Logistic regression revealed a statistically significant association between sex and clustering membership. This finding should be interpreted with caution and validated in independent cohorts. It raises the possibility of sex-specific influences - including genetic or epigenetic mechanisms and differences in susceptibility to environmental exposures or infectious agents - that merit further investigation. However, several factors could inflate this association: limited sample sizes in some strata, sensitivity to how clusters are defined, residual confounding (e.g., by age, period, mobility, or sociodemographic context), and multiple testing. We therefore treat the finding as hypothesis-generating.

### Strengths

This study’s main strength is its nationwide, population-based design, using high-coverage data (> 90%) from the SCCR and NCR across all Swedish municipalities. It also benefits from a long study period (1992–2017) and the ability to confirm the municipality of residence at diagnosis for all cases and at birth for over 99.5% of cases. Additionally, Sweden has harmonized cytogenetic and genetic diagnostics, and karyotypes have been centrally reviewed annually since 1996.

The use of both the CK-Test and K-Test strengthens the analysis. The CK-Test accounts for multiple testing and population shifts, ensuring that clustering patterns are not due to the underlying population distribution or chance. The K-Test assesses significance at prespecified space–time thresholds, providing a broader view of clustering dynamics. Combining these tests improves accuracy and reliability by identifying robust patterns across multiple space–time thresholds.

### Limitations

Exact street addresses were not accessible due to restrictions under the approved ethics protocol. Residential data were only available at the municipality level, which may introduce spatial imprecision and potential ecological or positional misclassification: cases within the same municipality can be geographically far apart, particularly in larger municipalities. This aggregation can both dilute short-range clustering and make the results sensitive to the chosen spatial bands. Likewise, birth dates recorded at the month level preclude sub monthly temporal thresholds and limit resolution of very short latency processes.More granular address-level geocoding and exact dates would have enabled finer spatial/temporal bands, evaluation of very short thresholds, and modeling frameworks that account for population heterogeneity and mobility, thereby refining estimates of critical space–time scales.

## Conclusion

We were able to detect space–time clustering in the genetic BCP-ALL subtype HeH, suggesting the presence of an underlying common etiologic factor. However, we did not observe space–time clustering in the overall BCP-ALL cohort, nor in the T-ALL cohort or the *ETV6::RUNX1* cohort. Further studies on genetically characterized ALL subtypes in larger cohorts are needed to confirm these findings and to gain a better understanding of the underlying mechanisms and causes of clustering.

## Supplementary Information

Below is the link to the electronic supplementary material.

Supplementary Material 1
