# Supplementary material for: Space–time clustering of childhood high hyperdiploid B-cell precursor acute lymphoblastic leukemia: a nationwide Swedish study
Source: Eur J Epidemiol. 2026 Jan 12;41(2):149–60. doi: 10.1007/s10654-025-01323-9 (PMC12975832; doi:10.1007/s10654-025-01323-9)
Supplement: Supplementary file 1 — Supplementary Material 1 [file 10654_2025_1323_MOESM1_ESM.docx]

**Statistical methods**

*Knox test*

The Knox test [1] is a statistical method used to detect space-time clustering by analyzing the spatial and temporal distribution of events. It compares the observed number of close pairs of events (based on specified spatial and temporal thresholds) to the expected number of close pairs in a random distribution.

Under the null hypothesis of no space–time interaction, the expected number of close pairs in space and time, $E(X)$, is calculated as:

$$E\left( X | N_{t},N_{s} \right)=\frac{N_{t}N_{S}}{N},$$

where $N_{t}$ is the number of close case pairs in time, $N_{s}$ is the number of close pairs in space, and $N$ is the number of distinct pairs of cases, $N=\frac{n\left( n-1 \right)}{2}$, where $n$ is the number of cases.

A prerequisite for using the Knox test is that $X$ follows a Poisson distribution. Barton and David [2] demonstrated that this assumption holds only when $N_{t}$ and $N_{s}$ are small relative to $N$. To address this, they proposed an empirical formula for the variance of $X$:

$$V\left[ X | N_{t},N_{s},N_{2s}, N_{2t} \right]=\frac{N_{s}N_{t}}{N}+\frac{4N_{2s}N_{2t}}{n(n-1)(n-2)}+\frac{4\left[ N_{s}\left( N_{s}-1 \right)-2N_{2s} \right][N_{t}\left( N_{t}-1 \right)-2N_{2t}]}{n(n-1)(n-2)(n-3)}-\left( \frac{N_{s}N_{t}}{N} \right)^{2},$$

in addition to $N_{t}$ and $N_{s}$, $N_{2t}$ and $N_{2s}$ represent the second-order pair terms, which account for the number of times two adjacencies are contiguous in space and time. These second-order terms must also be considered to more accurately calculate the variance.

*An Unbiased Knox Test, K-Test*

The original Knox test is biased in the presence of population shifts because it assumes that the spatiotemporal distribution of cases is homogeneous across the study area. However, population shifts such as migration or changes in population density, can introduce bias that affects the results of the test. For instance, if a large number of people move into an area during a certain period, the Knox test may detect clustering simply because the spatial distribution of cases has changed, not because of an underlying pattern of disease transmission. This bias occurs because the test does not account for these changes in population dynamics, leading to false positives or an overestimation of clustering.

To calculate the unbiased version of the original Knox test, we followed the methodology that was proposed by Kulldorf and Hjalmars [3] to avoid population shift biases. The unbiased Knox test is referred to as the K-Test in in this Supplementary Material and also in the Main section.

The key notion of the K-Test is the normalized excess in the number of close pairs, which is computed by subtracting the expected number of cases $E(X|N_{t},N_{s})$ from the observed number of close pairs (X) and dividing the difference by the variance:

$$N\left( X \right)=\frac{X-E(X|N_{t},N_{s})}{V[X|N_{t},N_{s},N_{2s},N_{2t}]},$$

where $X$ is the observed number of close pairs, $E\left( X | N_{t},N_{s} \right)$is the expected value of $X$, and $V[X|N_{t},N_{s},N_{2s},N_{2t}]$ is the variance of the random variable.

To account for population shifts, we reference $N(X)$ to the underlying population-at-risk surface. The K-Test was implemented as follows:

1. Null Sampling. Generate $M$ synthetic datasets by randomly sampling from the population-at-risk, preserving the marginal distribution of residential locations and key demographic variables; each synthetic dataset matches the observed sample size.
2. Specify threshold. Pre-specify a spatio-temporal threshold that defines a “close pair”.
3. Compute the statistics. For the observed data and for each synthetic dataset, compute the normalized excess of close pairs, $N(X)$, at the chosen threshold.
4. Evaluate the significance. Let $N_{obs}$ be the statistic from the observed data and $N_{1}, \ldots, N_{m}$ be the statistics from the synthetic dataset. The Monte Carlo p-values is:

$$p=\frac{1+ \sum_{m=1}^{M} \left\{ N_{obs}\leq N_{m} \right\}}{M+1},$$

i.e., the rank of $N_{obs}$ within the null distribution divided by $M+1$.

The K-Test was used here to improve the detection of space-time clusters by accounting for changes in population density. This adjustment is particularly useful in epidemiological studies, where population shifts can confound the results of the traditional Knox test, it helps to mitigate the bias introduced by variations in population density.

The main disadvantage of the K-Test is that it calculates the p-value for only one selected pair of spatial and temporal thresholds, making it difficult to identify the specific spatio-temporal pair to search for an interaction. In practice, a grid of spatio-temporal thresholds is defined, and multiple K-Tests are performed simultaneously, which introduces the problem of multiple testing. Adjusting the p-values using the Bonferroni-type method penalizes the tests too strictly, as they are not independent. To account for both population shifts and multiple testing, the Unbiased Combined Knox Test is used to provide more accurate p-values. The Unbiased Combined Knox test is referred to as the CK-Test in this Supplementary Material and in the main section.

*An Unbiased Combined Knox Test, CK-Test*

The CK-Test estimates the normalized number of close pairs across a grid of prespecified spatial and temporal thresholds. For the observed dataset and for each synthetic dataset, we compute the statistic at every grid point and retain the maximum value. Significance is then assessed by comparing the observed maximum to the distribution of maxima from the synthetic datasets (a “best-vs-best” comparison), which provides a multiplicity-adjusted Monte Carlo p-value.

The procedure is similar to the adjustment done for the K-Test:

1. Null sampling. Generate $M$ synthetic datasets by sampling from the population at risk, preserving the distribution of residential locations and key demographic variables; each synthetic dataset matches the observed sample size.
2. Specify thresholds. Define the grid of prespecified spatial and temporal thresholds $\{(\Delta t_{i}, \Delta s_{j})\}$ that determine a “close pair”.
3. Compute the statistics. For the observed data and for each synthetic dataset, compute the normalized excess of close pairs, $N(X)$, at the chosen thresholds.
4. Take the maxima. Record $N_{obs}^{max}=\max_{i,j} N(X_{obs};\Delta t_{i},\Delta s_{j})$ and, for each synthetic dataset $m=1,\ldots, M, N_{m}^{max}=\max_{i,j} N(X_{m};\Delta t_{i},\Delta s_{j})$.
5. Evaluate the multiplicity-adjusted p-value:

$$p=\frac{1+ \sum_{m=1}^{M} \left\{ N_{obs}^{max}\leq N_{m}^{max} \right\}}{M+1}.$$

The CK-Test produces p-values adjusted for both multiple testing and population shifts. It can be viewed as a global test that assesses whether clustering exists in the data, but it does not directly identify the most indicative pair of spatio-temporal thresholds. In this study, we first test for global clustering in the analyzed cohort. If the CK-Test produces significant results, we then perform individual K-Tests for all combinations of space-time thresholds and examine the most indicative combinations without adjusting p-values. Therefore, the p-values in Tables 2 and 3 do not exactly match.

**Population sampling**

*Procedure:*

1. Sample the year of birth with replacement according to the population distribution during the study period.
2. Sample with replacement municipality of residence at birth according to the population distribution during the previously simulated year.
3. Sample with replacement month of birth according to the distribution of months of birth frequency during the study period.
4. Sample the time-to-diagnosis using a Piecewise Linear Nonparametric CDF estimate [4] of time-to-diagnosis variable using the data.
5. Using the year of the simulated date of diagnosis, sample with replacement municipality according to the population distribution during that year.

Population sampling is used to adjust K-Tests and CK-Tests for potential biases arising from population shifts. Steps 1 through 3 are required to construct the dataset for clustering by place and date of birth. All five steps are necessary to create synthetic datasets for clustering by place and date of diagnosis.

**Machine learning clustering algorithm, DBSCAN**

After obtaining the most indicative space–time threshold pair, we employed the DBSCAN (Density-Based Spatial Clustering of Applications with Noise) machine learning algorithm to visualize and obtain clusters using optimal thresholds. The DBSCAN is a commonly used machine learning clustering algorithm that groups data points into clusters based on their density [5].

In DBSCAN, two points are considered neighbors if their distance is less than or equal to the specified radius $\varepsilon$. We modified the method such that two points are considered neighbors if their spatial (geographical) distance is smaller or equal to the specified spatial threshold and their temporal distance is smaller or equal to the specified temporal threshold. The second parameter, $minPts,$was set to 2.

**General procedure**

The general procedure for identifying a space-time clustering in this study is as follows:

1. Identify clinically relevant critical spatio-temporal thresholds.
2. Perform a CK-Test, which produces p-values adjusted for both multiple testing and population shifts, to determine if there is a space-time clustering in the analysed cohort and across all critical spatio-temporal thresholds.
3. If a space-time clustering is detected by the CK-Test, perform K-Tests to evaluate the distribution of p-values — adjusted for population shifts but not for multiple testing — across individual combinations of space-time thresholds.
4. Apply the DBSCAN machine learning method using the most indicative spatio-temporal pair.
5. Perform supplementary logistic regression analysis of clustered and non-clustered data.

**References**

1. Knox EG. The Detection of Space‐Time Interactions. Journal of the Royal Statistical Society Series C. 1964;13(1):25–9.

2. David FN, Barton DE. Two Space-time Interaction Tests for Epidemicity. Br J Prev Soc Med. 1966 Jan;20(1):44–8.

3. Kulldorff M, Hjalmars U. The Knox Method and Other Tests for Space-Time Interaction. Biometrics. 1999 Jun 1;55(2):544–52.

4. Kaczynski W, Leemis L, Loehr N, McQueston J. Nonparametric Random Variate Generation Using a Piecewise-Linear Cumulative Distribution Function. Communications in Statistics - Simulation and Computation. 2012 Apr 1;41(4):449–68.

5. Ester M, Kriegel HP, Sander J, Xu X. A density-based algorithm for discovering clusters in large spatial databases with noise. Inkdd 1996 Aug 2 (Vol. 96, No. 34, pp. 226-231).
